# Reference data on thickness and mechanics of tissue layers and anthropometry of musculoskeletal extremities

**DOI:** 10.1038/sdata.2018.193

**Published:** 2018-09-25

**Authors:** Erica E. Neumann, Tammy M. Owings, Tyler Schimmoeller, Tara F. Nagle, Robb W. Colbrunn, Benjamin Landis, J. Eric Jelovsek, Mike Wong, Joy P. Ku, Ahmet Erdemir

**Affiliations:** 1 Department of Biomedical Engineering, Cleveland Clinic, Cleveland, Ohio, USA; 2 Potrero Scientific, LLC, San Francisco, California, USA; 3 Department of Bioengineering, Stanford University, Stanford, California, USA

**Keywords:** Musculoskeletal system, Data publication and archiving, Biomedical engineering

## Abstract

Musculoskeletal extremities exhibit a multi-layer tissue structure that is composed of skin, fat, and muscle. Body composition and anthropometric measurements have been used to assess health status and build anatomically accurate biomechanical models of the limbs. However, comprehensive datasets inclusive of regional tissue anatomy and response under mechanical manipulation are missing. The goal of this study was to acquire and disseminate anatomical and mechanical data collected on extremities of the general population. An ultrasound system, instrumented with a load transducer, was used for in vivo characterization of skin, fat, and muscle thicknesses in the extremities of 100 subjects at unloaded (minimal force) and loaded (through indentation) states. For each subject, the unloaded and loaded state provided anatomic tissue layer measures and tissue indentation response for 48 and 8 regions, respectively. A publicly available web-based system has been used for data management and dissemination. This comprehensive database will provide the foundation for comparative studies in regional musculoskeletal composition and improve visual and haptic realism for computational models of the limbs.

## Background & Summary

Musculoskeletal extremities are composed of layers of skin, fat, and muscle surrounding the bone. These layers create a composite architecture and likely dictate the mechanics of the region as a result of individual tissue mechanical properties and anatomy. In return, they will determine the mechanical capacity to support the skeleton and protect against mechanical loading. Injuries to the extremities are common and vary in damage intensity from minor bruises of civil life, to pressure ulcers^1^ in the clinical setting, to blast injuries and gun-shot wounds on the battle field^2,3^. These injuries often require surgery, therapy, and rehabilitation to restore normal function and improve the patient's quality of life post-injury. Surgical outcomes may improve through medical training using surgical simulations. To accomplish this, simulation models should have high fidelity and represent actual tissue characteristics between skin, fat, and muscle layers.

Building reliable models of layered tissues requires acquisition of anatomical and mechanical properties of these tissue layers. Several separate studies have characterized the mechanical behavior of skin^4^, fat^5^, and muscle^6^. However, properties of each tissue were not collected from the same specimen or subject, therefore, mechanics could only be inferred in an aggregate manner rather than a relational definition. To capture variations within the diverse population at the organ and tissue levels, geometric and mechanical variations of the musculoskeletal soft tissue surrounding the extremities should be captured in a subject-specific manner.

**Figure 1:**
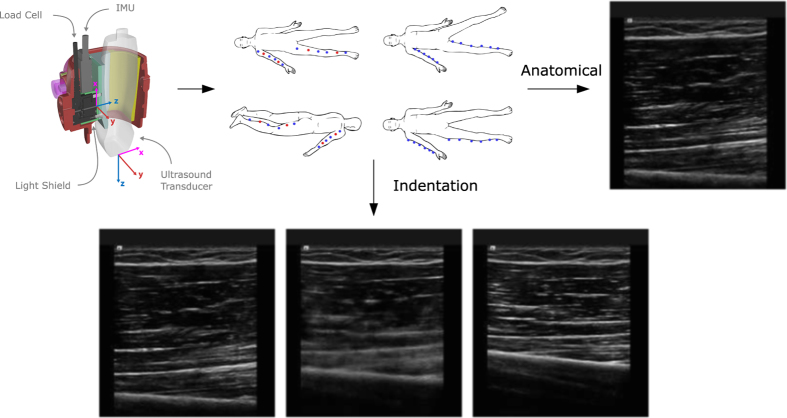
Data collection flowchart. Instrumented ultrasound is used to image 48 anatomical sites (all dots) and 8 indentation sites (red dots only).

The goal of this study was to develop a database of anthropometric measurements, soft tissue thicknesses, and loading response, in this case indentation, of human musculoskeletal extremities collected in vivo (Fig. 1). In addition, the reliability of thickness measurements (skin, fat, and muscle), collected via ultrasound images, was investigated. This dataset is the first portion of a larger project, aiming to develop reference finite element representations of the non-linear mechanics for multi-layer tissue structures, however, the data may be used for different applications. By analyzing a diverse sample of the healthy population, this dataset may provide insight into the most appropriate and accessible regions for appendicular body composition analysis^7–9^. Similarly, the wide array of regional images could be used to train musculoskeletal ultrasound sonographers. Indentation mechanics from this dataset may also be used as a healthy control group for comparison to a diseased population. For example, Makhsous *et al*. compared able-bodied subjects to individuals with chronic spinal cord injury to investigate changes in soft tissue stiffness^10^. Finally, this dataset may be used to design clothing, protective equipment^11^, or sockets for artificial limbs^12^.

## Methods

### Subjects Overview

One hundred adult subjects (50 male, 50 female) were recruited to participate in this study, which was approved by the Cleveland Clinic Institutional Review Board and the Human Research Protection Office of the U.S. Army (USAMRMC ORP HRPO). Informed consent was obtained from all subjects. Subject demographics, activity level, height, and mass were recorded for each participant in the study (Table 1). Self-reported activity level was recorded as one of five pre-defined categories, defined by the self-reported number of steps taken per day, frequency of exercise, and intensity of activity (Table 2). Subjects wore lightweight clothing, either shorts and a t-shirt or a hospital gown, for the duration of data collection.

### Anthropometric Measurements

**Figure 2:**
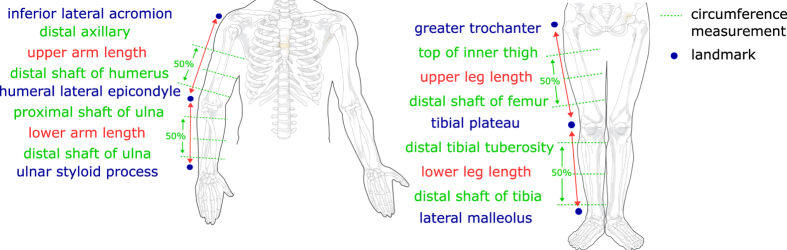
Arm and leg landmark and circumference measurement sites. Image modified from the original, which was released into the public domain. Location of central circumference measurements were at the mid-point between distal and proximal measurement locations (marked by 50%).

Anthropometric data, including length and circumference, were collected on the right leg and arm. Limbs were divided into upper and lower segments, where segment length and three circumferences (proximal, central and distal) were to be measured. Using a washable marker, reference marks were made on landmarks of the extremities (Fig. 2) while the subject was lying supine. Segment length and circumference measurements were recorded, along with the distance of each circumference location to the nearest superior landmark (Fig. 2). Single measurements were recorded to the nearest millimeter by the same investigator, using a cloth tape measure. All subjects had complete sets of anthropometric measurements.

### Ultrasound Image Acquisition

**Figure 3:**
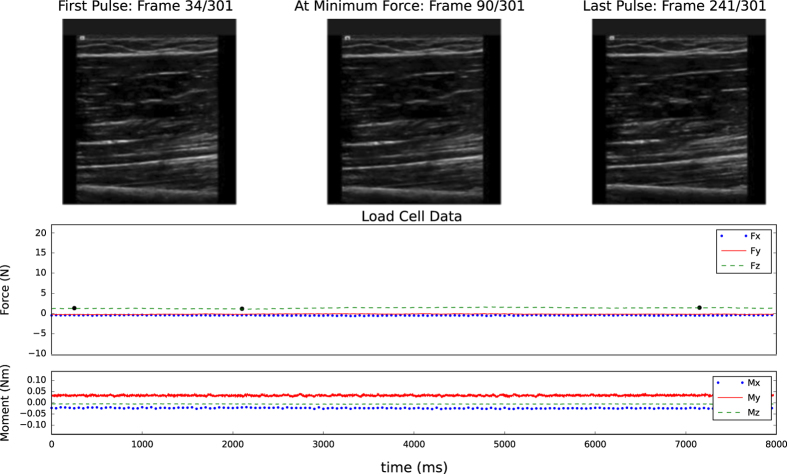
Sample anatomical images with corresponding forces and moments for the upper arm anterior central region of a 35-year-old male subject. Forces and moments are represented in the probe coordinate system (Fig. 1).

**Figure 4:**
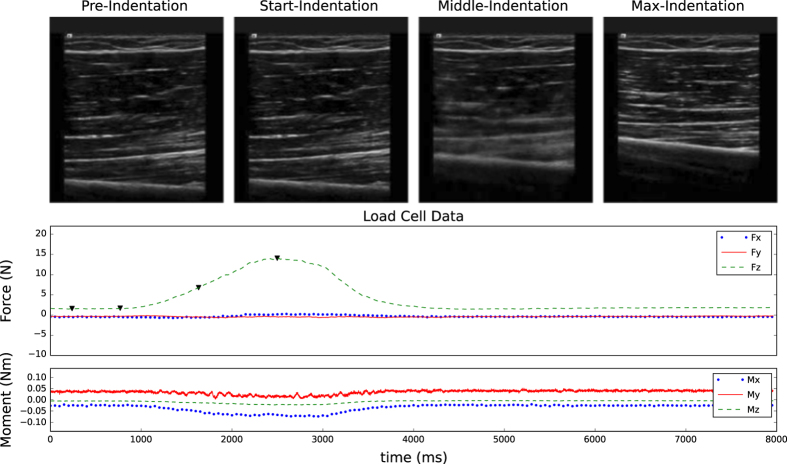
Sample indentation images with corresponding forces and moments for the upper arm anterior central region of a 35-year-old male subject. Forces and moments are represented in the probe coordinate system (Fig. 1).

A custom load transducer instrumented ultrasound device was used to image skin, fat, and muscle layers in the extremities (Acuson S3000, Siemens, USA). The ultrasound probe (14L5 or 9L4) was attached to a 6-axis load transducer (Nano25, ATI Industrial Automation, USA), to ensure that the bulk tissue was minimally loaded (force magnitude <2.2 N) during anatomical imaging (see Fig. 3 for sample data). An automated data association process was used to synchronize force data to the ultrasound images. Images were oriented along the length of the limbs at the 12 circumferences that were described previously, in the anterior, posterior, medial, and lateral directions. Subjects remained in the supine position for image capture, with the exception of posterior image capture where they laid in the prone position. The probe was aligned to the appropriate circumference using the proximal edge, centerline, and distal edge for proximal, central and distal imaging locations, respectively. In addition to the anatomical imaging, indentation response was also collected for the anterior and posterior central locations of each segment (8 total trials) (see Fig. 4 for sample data). Depth of imaging was adjusted to obtain the highest resolution while still viewing the entire thickness of soft tissue to the bone boundary (note: targeted bone varies, see Table 3). Gain was also adjusted to improve signal and obtain improved distinction between soft tissue layers. Focus was typically positioned halfway through the depth of the soft tissue to bone, however, after visual inspection, it did not significantly impact image sharpness or quality for thickness measurements.

### Thickness Analysis

Ultrasound images were analyzed using a custom Python script (https://www.python.org/) to interactively measure skin, fat and muscle thicknesses for both the anatomical and indentation trials. A graphical user interface was developed to automatically extract the ultrasound frame corresponding to the minimum force for each anatomical trial and for the entire duration of the compression portion of each indentation trial. After applying a central moving average filter (150 points on each side of data point) to the force magnitude, the start of indentation was determined by the time-point when the slope (working backwards from the peak force) transitioned from greater than 1 N/sec to less than 1 N/sec. For thickness measurement, the analyst placed four red circular markers on each tissue boundary: transducer/skin, skin/fat, fat/muscle, and muscle/bone (see “Technical Validation” section below for repeatability). The thicknesses and corresponding probe forces and moments were recorded and stored in a file with an XML format.

### Code Availability

The code for data processing and image analysis can be found at the project site (https://simtk.org/projects/multis); specifically, in the source code repository of the project website (https://simtk.org/scm/viewvc.php/?root=multis). In addition, a frozen download package of the thickness analysis software has been released (https://simtk.org/frs/?group_id=1032). All scripts were developed in Python 2.7. The DICOM and npTDMS libraries were used to read the ultrasound and data files, respectively. Additional Python libraries were used to develop the analysis scripts, where most can be easily installed using packages such as Anaconda (“conda install” command) (https://docs.anaconda.com/anaconda/).

## Data Records

File structure and naming conventions were followed to simplify the raw and derivative data organization, making it easier to browse and query the disseminated data. Each subject has a separate root directory, named with the de-identified subject ID, that has a folder structure as follows:

Raw data:

1. *Data –* Probe load and orientation data in raw form and transformed (ultrasound probe tip coordinate system with weight compensation) (.tdms)
2. *Configuration* – Contains the sensor and state files used during data collection for each data file (.cfg). The sensor and state files provide the structure and description of the binary data file (.tdms) for the raw and transformed data, respectively. Also contains the subject configuration file [.cfg and .xml, which provides the file locations (state.cfg, sensor.cfg, data.tdms) of each data collection site and de-identified subject information (demographics and anthropometrics)].
3. *Ultrasound* – DICOM image volumes for each trial (.IMA)

Derivative data:

File Association:

1. *DataOverview* – Provides a summary figure of each individual trial, including ultrasound frames throughout the trial with corresponding forces and moments in the ultrasound probe tip coordinate system.
2. *FileAssociation* – Provides a figure for each individual trial showing the raw and time adjusted analog signal between the data (.tdms) and ultrasound (.IMA) systems for time synchronization.
3. *TimeSynchronization* – Contains an XML for each trial that stores the time shift (dT, measured in milliseconds) between the data (.tdms) and ultrasound (.IMA) systems.

Thickness Analysis:

- *TissueThickness* – Sub-directory that contains all thickness analysis.
- o *UltrasoundManual –* Manual identification of tissue boundaries from ultrasound imaging of each trial (XML)
- ■ *ThicknessPNG –* Provides image of the first ultrasound frame that was analyzed (corresponding to minimum force or initiation of indentation, for anatomical and indentation trials, respectively) and a plot of force magnitude vs. thickness (skin, fat, muscle, and total).

Raw and derivative data files exist for each trial with the following root naming convention:

*“Experiment Run Number”_”Subject ID”_”Limb Segment”_”Location”_”Test Type”-”Trial Number Index”*

- Experiment Run Number: 3-digit number that gets auto-incremented for each tdms file (order in which data are collected for each specimen).
- Subject ID: 3-digit number that gets assigned to each subject in the order that they were collected.
- Limb Segment: Two letter abbreviation for the trial segment, UA = upper arm, LA = lower arm, UL = upper leg, LL = lower leg
- Location: Two letter abbreviation describing the imaging location. First letter – (A)nterior, (P)osterior, (M)edial, (L)ateral. Second letter – (P)roximal, (C)entral, (D)istal.
- Test Type: I = indentation, A = anatomy
- Trial Number Index: auto-incremented for each trial, i.e. if the location is tested more than once the subsequent data files will be −2, −3, etc.

Additional characters are appended to the root name for further data descriptions. Example file names for a single trial include:


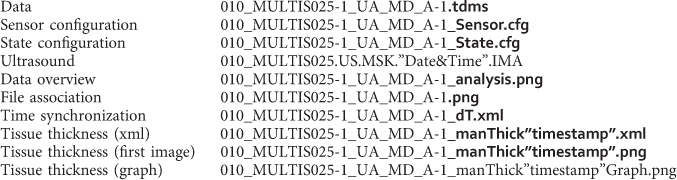


Experimental specifications and detailed information on the infrastructure for this project are publicly accessible and can be found on the SimTK project website (https://simtk.org/projects/multis).

## Technical Validation

To quantify force during anatomical and indentation trials, the load was measured at the ultrasound probe tip by removing the weight of the probe and applying a transformation matrix from the load cell origin to probe tip. Two validation experiments were performed to test the accuracy of the weight compensation and probe tip transformation. After performing gravity compensation, the resultant force was measured in multiple, random orientations. The probe was handheld and kept still for each trial. In the first experiment, no load was applied to the probe tip. In the second experiment a mass was suspended via a string from a 3D printed ultrasound probe tip. The resultant force of twelve orientations with zero applied load was 0.02±0.01N. The resultant force of twelve orientations with a 4.82N suspended weight was 4.83±0.03N.

A limitation of ultrasound imaging is that the tissue interface boundary of the muscle fascia that may appear as a false bone boundary. Therefore, prior to thickness analysis, the analyst was guided by an ultrasound radiologist on the appropriate landmarks for accurate identification of tissue interfaces. For each subject, notes were recorded for locations that did not contain soft tissue visualization down to the bone (see project wiki at https://simtk.org/plugins/moinmoin/multis/Subjects). In addition, data cleaning was done by inspecting anthropometric measurements and thickness results that were outside of two standard deviations from the location-specific mean of the entire dataset. Notes were recorded on the project wiki (noted above) in cases where correlations to location circumference or subject BMI were un-identifiable. Overall, there were 30 subjects with complete anatomical thickness analysis datasets. Lower leg medial proximal and central locations were responsible for 62 and 45 missing bone boundaries of the anatomical trials, respectively. Eighty-three subjects were missing thickness analysis at less than 3 anatomical locations. In addition, there were 90 subjects with complete indentation thickness analysis datasets. Again, detailed notes for each subject are included on the project wiki (noted above) and along with the data distribution.

**Figure 5:**
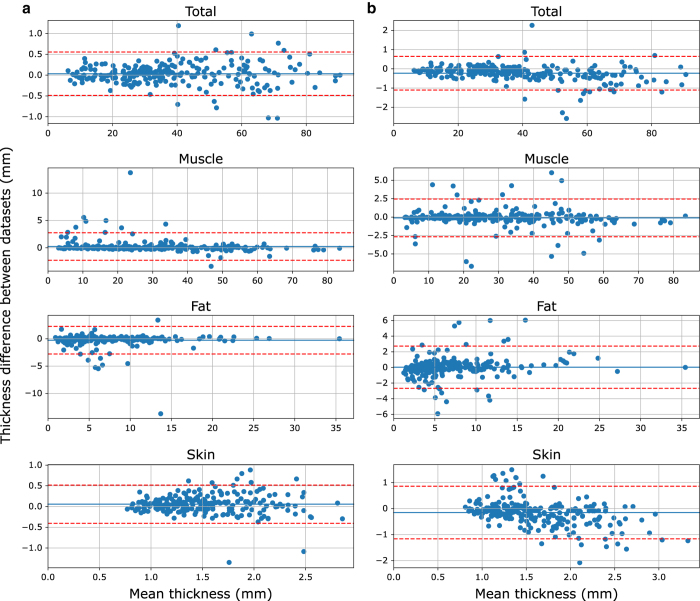
Bland-Altman plots showing repeatability for the total soft tissue, muscle, fat, and skin thicknesses of anatomical trials. (**a**) Intra-observer differences and (**b**) inter-observer differences. The solid blue line represents the mean difference and the dotted red lines indicate two standard deviations from the mean.

**Figure 6:**
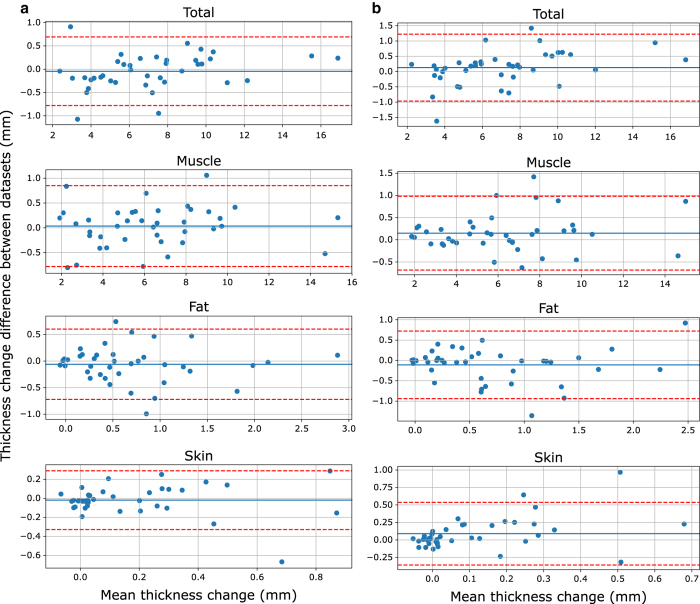
Bland-Altman plot showing the repeatability for the total soft tissue, muscle, fat, and skin thickness changes of indentation trials. (**a**) Intra-observer differences and (**b**) inter-observer differences. The solid blue line represents the mean difference and the dotted red lines indicate two standard deviations from the mean.

Thickness measurement repeatability was evaluated by using data from all 48 anatomical trials and all 8 indentation trials for 5 randomly selected patients (with complete datasets). Intra-observer variability was completed at least one week apart and the analyst was blinded to their previous thickness results. Inter-observer thickness variability was measured between two analysts who were blinded to thickness results of the other analyst. Mean absolute differences (±SD) for anatomical trials were 0.16±0.17, 0.49±1.19, 0.48±1.19, and 0.19±0.19 mm (intra-observer) and 0.36±0.38, 0.78±1.10, 0.65±1.11, and 0.32±0.38 mm (inter-observer) for skin, fat, muscle, and total tissue thicknesses, respectively (Fig. 5). Thickness change from beginning of indentation to peak force was used as a metric for comparison of indentation trials. Mean thickness change absolute differences (±SD) for indentation trials were 0.10±0.12, 0.24±0.24, 0.32±0.25, and 0.29±0.23 mm (intra-observer) and 0.15±0.19, 0.29±0.32, 0.31±0.31, and 0.42±0.37 mm (inter-observer) for skin, fat, muscle, and total tissue thickness change, respectively (Fig. 6).

As noted above, ultrasound images are difficult to interpret and require training. While less than 4% of each thickness measurement (skin, fat, muscle, and total) at the locations analyzed had intra-observer differences outside two standard deviations from the mean, outliers were present and the standard deviation of fat and muscle layers was larger than anticipated. The total soft tissue and skin thickness measurements have a few outliers, however the magnitude of the differences can be explained by a blurry transducer/skin boundary point (~1 mm maximum) in most cases, where vertical image resolution (range: 0.015–0.19 mm/pixel) limits the analyst. A second source of error related to the resolution variability is the topology changes of the tissues, especially skin, within the ultrasound images. Inexperienced analysts may incorrectly define the tissue boundaries, again highlighting the importance of training. When looking at the fat and muscle thickness differences beyond two standard deviations from the mean, the hyperechoic fat/muscle boundary may not appear as bright due to probe direction relative to the muscle fascia or there may be fibrillar structures within the fat layer that appear as false fat/muscle boundaries. Indentation repeatability was similar between intra- and inter-observer differences, which is likely due to clarity of the ultrasound images at the central regions where the muscle bellies are located. These observations suggest that improved image acquisition or additional training of the analyst may improve the repeatability of distinguishing between tissue layers, however the total soft tissue repeatability is within an acceptable range. The inter-observer variance is higher than the intra-person variance, which likely can be contributed to the experience of the analyst. In fact, skin thickness reaches an unreasonably high error in reference to the thickness (Fig. 5). This error increase is due to incorrect boundary definition from the inexperienced analyst. One analyst was responsible for analyzing all 100 subjects in this study, while the other had only met with the radiologist and been trained through use of example images. The inter-observer variance is expected to decrease over time as the analysts gain experience and approach the 'true' thickness value. These differences highlight the difficulty of ultrasound image analysis and importance of quality image acquisition. This data set provides all of the raw ultrasound images, giving other researchers the opportunity to conduct their own measurements using manual or automated thickness measurement methods, if desired.

## Usage Notes

All raw and derivative data can be found on the dynamic data management site: https://multisbeta.stanford.edu/. In addition, a static version (frozen at the time of publication) of the data is provided (Data Citation 1). The data is accessible to anyone who registers for an account on the site. Registration only requires a valid e-mail address and enables the project team to understand data usage statistics in an aggregated manner. The account will need approval from a project administrator, who is notified when a new account is created. After approval, all uploaded data are accessible at any time along with the data querying feature, which allows individuals to segment and filter the data according to the 43 metadata fields provided (e.g., age, gender, activity level, etc.).

Data analysis was completed using Python libraries. All scripts used to collect and summarize the data can be found on the project SimTK source code repository (https://simtk.org/scm/viewvc.php/app/?root=multis). In addition, a download package of the thickness analysis software used for this study has been released (https://simtk.org/frs/?group_id=1032). The data analysis workflow and specifications are described in detail on the data analysis wiki page (https://simtk.org/plugins/moinmoin/multis/Specifications/DataAnalysis).

## Additional information

**How to cite this article**: Neumann, E. E. *et al*. Reference data on thickness and mechanics of tissue layers and anthropometry of musculoskeletal extremities. *Sci. Data* 5:180193 doi: 10.1038/sdata.2018.193 (2018).

**Publisher’s note**: Springer Nature remains neutral with regard to jurisdictional claims in published maps and institutional affiliations.

## Supplementary Material



## Figures and Tables

**Table 1:** Subject demographics data key.

**Data Category**	**Description**	**Key**
Age	Subject's age in years	N/A
Gender	Subject's gender	0 – Male
		1 – Female
Ethnicity	Subject's ethnicity	0 – Hispanic or Latino
		1 – Not Hispanic or Latino
Race	Subject's race	0 – White
		1 – Black or African American
		2 – American Indian or Alaska Native
		3 – Asian
		4 – Native Hawaiian or Other Pacific Islander
Activity Level	Subject's activity level or lifestyle	0 – Extremely inactive
		1 – Sedentary
		2 – Moderately active
		3 – Active
		4 – Extremely active
Height	Subject's height in centimeters	N/A
Mass	Subject's mass in kilograms	N/A

**Table 2:** Self-reported activity category guidelines.

**Activity Level**	**Description**
Extremely inactive	Limited mobility or complete bedrest
	Unable to perform activities of daily living^a^
Sedentary	Desk worker with little or no exercise
	Activities of daily living^a^ only
	Less than 30 minutes of light activity per day
	Under 5,000 steps per day
Moderately active	Activities of daily living^a^ plus:
	Exercise 3–4 days per week for 1/2 – 1 h per day at moderate intensity or
	Additional daily activities (brisk walking, biking, raking leaves, swimming, dancing, water aerobics), or
	5,000 – 10, 000 steps per day
Active	Activities of daily living^a^ plus:
	Exercise5-7 days per week for 1–2 h per day at high intensity activities (aerobics, jogging, hockey, basketball, fast swimming, fast dancing), or
	10, 000 – 12, 500 steps per day
Extremely active	Activities of daily living^a^ plus:
	Exercise daily for 2+ hours per day at moderate-high intensity (aerobics, jogging, hockey, basketball, fast swimming, fast dancing), or
	Competitive athlete, military, fitness trainer, or
	Greater than 12, 500 steps per day
^a^Activities of daily living include: shopping, cleaning, watering plants, taking out the trash, walking the dog, mowing the lawn, and gardening.	

**Table 3:** Targeted bone for regions captured with ultrasound imaging.

**Extremity**	**Positions**	**Targeted Bone**
Upper Arm	All	Humerus
Lower Arm	Anterior, Posterior, Lateral	Radius
	Medial	Ulna
Upper Leg	All	Femur
Lower Leg	Anterior, Lateral, Medial	Fibula
	Posterior	Tibia
